# Exploring the risks of phage application in the environment

**DOI:** 10.3389/fmicb.2013.00358

**Published:** 2013-11-29

**Authors:** Sean Meaden, Britt Koskella

**Affiliations:** College of Life and Environmental Sciences, University of ExeterPenryn, UK

**Keywords:** phage therapy, antibiotic resistance, coevolution, phage resistance, microbial communities

## Abstract

Interest in using bacteriophages to control the growth and spread of bacterial pathogens is being revived in the wake of widespread antibiotic resistance. However, little is known about the ecological effects that high concentrations of phages in the environment might have on natural microbial communities. We review the current evidence suggesting phage-mediated environmental perturbation, with a focus on agricultural examples, and describe the potential implications for human health and agriculture. Specifically, we examine the known and potential consequences of phage application in certain agricultural practices, discuss the risks of evolved bacterial resistance to phages, and question whether the future of phage therapy will emulate that of antibiotic treatment in terms of widespread resistance. Finally, we propose some basic precautions that could preclude such phenomena and highlight existing methods for tracking bacterial resistance to phage therapeutic agents.

## INTRODUCTION

The selection for and subsequent evolution of antibiotic resistance in bacterial populations, both in the wider environment and during clinical treatment, presents a serious challenge to human health (Kåhrström, 2013). Although still controversial, it is increasingly clear that agricultural use of antibiotics has played a role in the continued selection for resistance genes and that the movement of these genes into pathogens of clinical relevance is possible (see Smith et al., 2009; van Cleef et al., 2010; Heuer et al., 2011; Zhu et al., 2013). Nosocomial environments also act as significant sources of antibiotic resistance and the transfer of antibiotic resistance genes into the agricultural environment has recently been demonstrated, suggesting the genetic exchange between environments works both ways (Price et al., 2012). As a result, alternative antimicrobial strategies are being sought. One such strategy is to utilize naturally occurring viral predators of bacteria: bacteriophages (phages). Lytic phages are capable of killing bacteria by invading and propagating within the host cell and then lysing open the cell to “burst” out, thus killing the bacteria. This is in contrast to temperate phages, which integrate into the genome of their hosts and can be transmitted vertically, serving as a refuge for phages in harsh environments (Svircev et al., 2011). The latter, although important to the ecology and evolution of bacterial populations, are not commonly considered for use as biocontrol and therefore will not be covered further in this review (but see Hyman and Abedon, 2010 for review of the effects of lysogeny on bacterial resistance). Despite being discovered as potential therapeutic agents over 80 years ago (d’Herelle, 1929), and their continual use in Russia and Georgia ever since (Kutter et al., 2010), few clinical trials of so-called “phage therapy” have been conducted in Western medicine (Wright et al., 2009; Sarker et al., 2012). Accordingly, no clinical phage therapy products are currently available in the West, and regulatory burdens may have dampened pharmaceutical interest, as years of research and clinical trials can cost millions of euros, presenting a formidable hurdle (Pirnay et al., 2011; Brüssow, 2012).

A more viable route to market has been provided by use of phages in agriculture and aquaculture (Jones et al., 2012; Martínez-Díaz and Hipólito-Morales, 2013). Recent review suggests that although phage therapy will not be the panacea that broad-spectrum antibiotics once were, phages could nonetheless play an important role in treating infections and maintaining food yields (Allen et al., 2013). Indeed, phage usage in agriculture has shown promise for treating numerous plant pathogens (Frampton et al., 2012), and some formulations of phages have been sold for large-scale environmental application (e.g., AgriPhage^TM^, Omnilytics). Likewise, the Food and Drug Administration in the USA has approved a product for the treatment of food products prior to market (Listshield^TM^, Intralytix) and classed the use of phages in this specific context as “generally recognized as safe” (Food and Drug Administration (FDA), 2013). Another fruitful avenue for phage therapy may be aquaculture, an industry that has increased globally by over 10-fold in the last 30 years (Food and Agriculture Organization [FAO], 2012). Microbial diseases represent a severe threat to aquaculture productivity; accordingly, phages capable of lysing pathogens such as *Flavobacterium psychrophilum*, the causative agent of bacterial cold-water disease, have been isolated and tested as therapeutic agents (Kim et al., 2010). Combined, the scope for phage therapy to fill food production and clinical niches left vacant by redundant antibiotics seems vast. However, whilst phage therapy as a biopesticide could prove a useful tool, it also presents a risk for repeating the mistakes made with overuse of antibiotics, and the subsequently high levels of evolved antimicrobial resistance observed both in the environment and in hospitals (Zhu et al., 2013). For example, it is unclear whether introducing high concentrations of diverse phage types into the environment will select for broad resistance, making future treatments less likely to succeed. Furthermore, the role of phage-mediated selection in shaping bacterial growth rates and virulence to their hosts remains poorly understood.

Despite a number of reviews highlighting the need for increased understanding of environmental perturbations from anthropogenic antibiotic input (Martínez, 2008; Ding and He, 2010;Allen et al., 2013), the impact of antibiotic use on microbial communities has rarely been taken into account when designing treatment or application. This is particularly surprising given the known natural importance of these chemicals in shaping competition among bacterial strains (D’Costa et al., 2011). As phages are also known to select for resistant bacteria (Buckling and Rainey, 2002) and to mediate competition among strains (Bohannan and Lenski, 2000; Koskella et al., 2012), the same risks should apply to this alternative treatment. Unfortunately, little is currently known about the effects of applying high titers of phages to natural microbial communities. Most importantly, it is possible that with uncontrolled application of phages in the environment the future efficacy of phage therapy in a clinical setting could be reduced – a mistake we cannot afford given the need for new antimicrobial therapies as a result of drug-resistant pathogens (Levy and Marshall, 2004). Phage therapy in agriculture could serve as a testing ground for clinical use (Stone, 2002; Levin and Bull, 2004). However, there could also be a conflict of interest if cross-resistance to phage treatments is possible and if these resistant bacteria can spread from agricultural to clinical settings, as has been observed for antibiotic resistance (van Cleef et al., 2010; Perry and Wright, 2013). If phage therapy treatments fail, or improper use of phages in the environment goes unchecked, the use of widespread phage biocontrol in agriculture could jeopardize the future of phage therapy in hospitals. Fortunately, our understanding of phage-mediated selection is growing at a rapid pace and a new era of genomic investigation should allow monitoring of microbial communities following phage therapy. In this review, we will discuss the status of the field of phage therapy and consider the implications of phage host range and bacterial resistance. We suggest that with a few precautions phage therapy may be effective for treating bacterial infections in agriculture, aquaculture, healthcare, food production and food safety.

## THE RISKS OF ANTIMICROBIAL USE IN AGRICULTURE

The argument against using antibiotics as standard agricultural practice, both to improve growth rates and prevent disease, is not new (Witte, 1998) and has been extensively reviewed previously (Singer et al., 2003). However, unequivocally demonstrating increased resistance as a consequence of agricultural usage has proved elusive (Perry and Wright, 2013). A wave of new data supporting both direct and indirect routes of antibiotic resistance genes between agricultural and human populations suggests a bidirectional zoonotic exchange (Price et al., 2012). For example, recent studies have found diverse and abundant resistance genes in manure prior to disposal in the environment (Zhu et al., 2013) and a high prevalence of resistance to multiple antibiotics in enterobacteria isolated from tomato farms (Micallef et al., 2013) and in bacteria from manure-amended soils (Popowska et al., 2012). Furthermore, methicillin-resistant *Staphylococcus aureus* (MRSA) rates in workers on swine farms have been shown to be higher than for the average population in both North America and Europe (Voss et al., 2005;Khanna et al., 2008; Smith et al., 2009; van Cleef et al., 2010). Finally, calves treated with antibiotics are also more likely to carry MRSA and there is a direct association between intensity of animal contact and human MRSA carriage (Graveland et al., 2010). A similar trend is seen in aquaculture where bacteria nearer to farms were found to have higher levels of antibiotic resistance than nearby coastal regions in Italy (Labella et al., 2013). The increasing number of studies supporting the hypothesis that environmental use of antibiotics has contributed to selection for antibiotic resistance suggests that non-prudent use of antibiotics in healthcare and agriculture may reduce the effectiveness of antibiotic strategies as an essential treatment for disease.

As an alternative to antibiotic use, the application of phages in agriculture is being trialed as a biopesticide to control plant pathogens of tomato (Jones et al., 2012), citrus (Balogh et al., 2008), and onion (Lang et al., 2007) among others (reviewed in Svircev et al., 2011). For example, *Erwinia amylovora* (the causative agent of fire blight) infections are affecting a number of crop species in orchards across North America and Europe (see Malnoy et al., 2012 for review). Although antibiotics have traditionally been employed to control this disease, the emergence of streptomycin resistant strains (McManus et al., 2002) and a desire to reduce antibiotic use in the environment has led to the use of phages as an alternative. Phage biocontrol clearly has the potential to control fire blight infections, as lytic phages have been isolated that are highly infective to the pathogen, but definitive field trials are currently lacking. Given the evidenced risks of movement of antibiotic resistance genes between agricultural to human pathogens, we should ask whether the large-scale application of phages is likely to repeat these past mistakes. Until appropriate studies are conducted, the subsequent consequences of applying phages in agriculture for the spread of antibiotic resistance, the evolution of the pathogen, and the community of microbes within the plants and soil remain unknown.

## DESIGN AND IMPLEMENTATION OF PHAGE THERAPY AND BIOCONTROL

**FIGURE 1:**
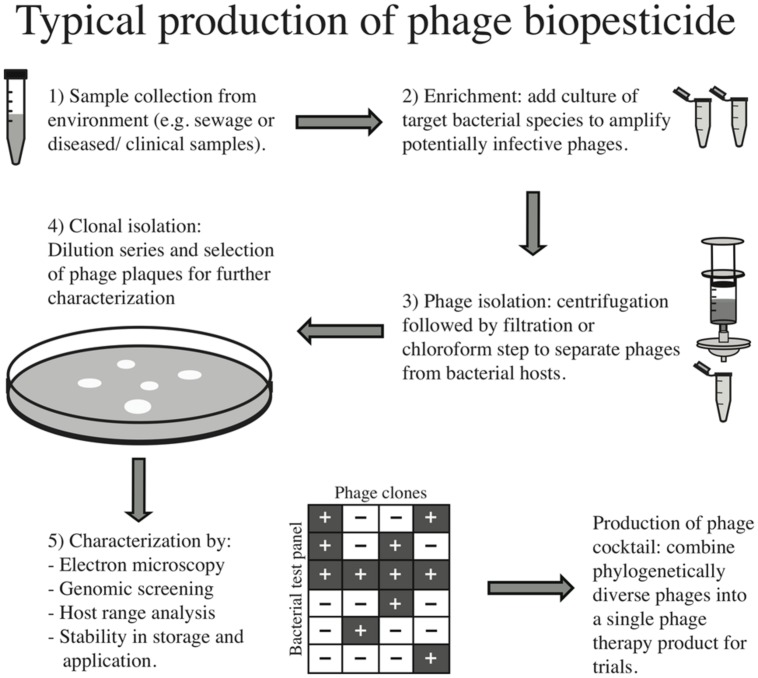
**Typical production of phage biopesticide**.

The process of preparing a phage therapy product for clinical use has been thoroughly described (Merabishvili et al., 2009; Gill and Hyman, 2010). **Figure 1** also describes this process for clinical and environmental samples. Briefly, environmental samples such as sewage or clinical samples from infected wounds are collected. The next step normally employs an “enrichment” process whereby the target bacterial species is added to the sample to increase the titer of phages infective to this strain. The sample is either filtered or chloroform is added to separate phages from bacteria, and individual phage “plaques” (i.e., the localized absence of bacterial growth in a lawn due to lysis) are chosen for further characterization. Transmission electron microscopy may be employed to assign family level phylogeny and genetic sequencing for finer scale taxonomic assignment, and screening of virulence factors is typically conducted. Other properties such as stability across a range of environmental conditions may be tested for optimal storage and production. Importantly, phage host range is typically tested to ensure the selected isolates have high efficacy against the pathogen of interest. However, this screening is most often done using a reference panel of laboratory stocks, rather than a large subset of bacteria from the local environment in which the phages will be applied, leading to a biased host range description. Therefore one way to reduce the possible community-level effects of applying phages would be to perform large-scale host range analyses across a biologically meaningful panel of isolates (i.e., those bacterial strains and species with which the phages are likely to interact once applied), as has been done successfully in the field of microbial ecology (Flores et al., 2011; Koskella and Meaden, 2013).

Once individual phages have been isolated and characterized, phage cocktails are produced by combining multiple, usually phylogenetically diverse, phages into one formulation. The idea behind these combined treatments is twofold: first, the use of multiple phages should increase the breadth of efficacy of the treatment to include most circulating strains of a pathogen; and second, the evolution of bacterial resistance should be slowed relative to single phage treatment. Whilst this approach could select for broadly resistant bacterial hosts, a number of studies suggest that broad resistance will carry a larger cost and therefore will not spread as rapidly (Bohannan and Lenski, 2000; Hall et al., 2012; Koskella et al., 2012). For example, strains of the plant pathogen, *Pseudomonas syringae*, evolved in the presence of three phage types were just as likely to evolve resistance against all three as those strains evolved in the presence of a single phage type. Furthermore, bacteria treated with multiple phages were no more likely to be cross-resistant to novel phages but were found to have paid a greater cost for their resistance than bacteria treated with a single phage type (Koskella et al., 2012). A similar result was observed for *Pseudomonas aeruginosa* strains treated with one versus four phages (Hall et al., 2012). This emulates the common practice of using combined antibiotic treatment to decrease the likelihood of evolved antibiotic resistance (Traugott et al., 2011; Vardakas et al., 2013). Overall, a greater understanding of the costs of resistance to phage predation and of synergistic effects among phages in controlling bacterial pathogens will allow for a more informed development and application of treatment, and ideally the prevention of widespread resistance.

## THE IMPLICATIONS OF EVOLVED RESISTANCE TO PHAGES

Despite the promise of many phage therapy trials, the use of phages to control bacterial pathogen begs the question: could the evolution of phage resistance mirror the evolution and spread of antibiotic resistance? Numerous studies have shown that natural phages are well-adapted to their local bacterial populations (Vos et al., 2009; Koskella et al., 2011) and that bacteria in turn adapt to resist their local phages (Kunin et al., 2008; Koskella, 2013). However, a recent review of phage resistance as a result of prolonged phage therapy (Ormälä and Jalasvuori, 2013) concludes that, as it is possible to isolate phages infective to bacteria from different geographical locations and evolutionary histories (e.g., Flores et al., 2011), long-term resistance need not be a concern as a diverse set of phages capable of infecting newly resistant strains will always be available. Local phage diversity is often high (Breitbart and Rohwer, 2005), so infective phages should be easy to isolate from just a few environmental samples. However, this parallels the problems of antibiotic discovery – the process from discovery to a useable product is arduous and expensive, so despite a ready source of infective phages few companies are investing in treatments (Brüssow, 2012). If bacterial resistance to phage infection emerges rapidly and production is slow, redundancy of treatments seems likely. As pointed out by Pirnay et al. (2011) a reactive phage therapy program that is capable of rapidly isolating, screening, and applying infective phages will be better placed to respond to phage resistance than the slow and expensive process of approval and licensing for each phage type.

Currently the maximum breadth of bacterial resistance to phage (i.e., the number of phage types a single bacterium is capable of resisting) remains largely unknown, as novel genera of phages are continually being discovered (Holmfeldt et al., 2013). For example, the ubiquitous marine bacterial clade SAR116 was thought to be so abundant as a result of escaping phage predation, but a recent finding shows that it is indeed infected by phages, and that these phages are likely to be the most abundant species on the planet (Kang et al., 2013). Our knowledge of phage ecology and evolution is still in its infancy; the exact mechanisms of infection, and in turn resistance, are often unknown and could be simultaneously diverse among strains and yet largely conserved across genera (Koskella and Meaden, 2013). There are a number of published cases of phages that are capable of infecting bacterial hosts across genera (**Table 1**), suggesting the potential for shared resistance mechanisms. Even if unlikely, evolved resistance to the few phage therapy products available to clinicians would severely impair treatment potential. This problem may be exacerbated by the more stringent control of phage products for clinical use, and thus the slow pipeline from isolation to delivery, relative to the approval of cocktails for use in agriculture. As such, rapidly responding regulation, like the measures in place for seasonal flu vaccines (Verbeken et al., 2012), could be a more effective way of countering phage therapy product redundancy.

**Table 1:** Examples of phages isolated from the environment capable of infecting across genera.

Author/s (year)	Genera infected	Number of genera	Pathogenicity
Koskella and Meaden (2013)	*Pseudomonas* and *Erwinia*	2	Crop pathogens
Lu et al. (2012)	*Lactobacillus* and *Weissella*	2	Endocarditis and bacteremia
Evans et al. (2010)	*Serratia* and *Pantoea*	2	NA
Bielke et al. (2007)	*Salmonella* and *Klebsiella* or *Escherichia*	2	Enterocolitis, pneumonia, urinary tract infections, and septicemia
Beumer and Robinson (2005)	*Sphaerotilus* and *Pseudomonas*	2	Pneumonia, urinary tract infections, septicemia, and wound infection
Thomas et al. (2002)	*Gordonia*, *Nocardia*, and *Rhodococcus*	3	Opportunistic pathogenesis
Jensen et al. (1998)	*Pseudomonas* and *Sphaerotilus* or *Escherichia*	2	Pneumonia, urinary tract infections, septicemia, wound infection, and enteric disease
Tilley et al. (1990)	Multiple genera within the *Micromonospora*	3	Respiratory infection


Finally, the combined use of phages and antibiotics has shown great promise due to the negative pleiotropic effects of phage resistance and antibiotic resistance. Experimental evolution has demonstrated that phages applied to populations of *Pseudomonas fluorescens* that had evolved antibiotic resistance reduced population densities to a greater degree than when applied to sensitive strains (Escobar-Páramo et al., 2012). In addition, combined treatment was shown to drastically hinder the evolution of bacterial resistance over time compared to antibiotic treatment alone (Zhang and Buckling, 2012). In poultry, the combination of phages and enrofloxacin resulted in lower mortality in infected birds than either treatment individually (Huff et al., 2004). Therefore, one potential step forward in controlling the spread of both antibiotic resistance and phage resistance in the environment and/or under clinical settings could be the carefully planned combination treatments of the two.

## PHAGE-MEDIATED ATTENUATION OF BACTERIAL VIRULENCE

A common refrain of phage therapy and biocontrol is that even if resistance does emerge, such resistance is likely to be costly, and as such would attenuate bacterial virulence in a eukaryotic host (Inal, 2003; Hagens et al., 2004). Phage resistance does seem to be correlated with a reduction in metabolic fitness both in the lab (Bohannan and Lenski, 2000; Koskella et al., 2012) and the soil (Gómez and Buckling, 2011), however the effect this may have on virulence in a eukaryotic host is largely unknown. Of the few examples, phage-resistant strains of *Yersinia pestis* have been shown to have attenuated virulence in a mouse model system, resulting in a significant increase in time to death, and in some cases complete attenuation (Filippov et al., 2011). The same phenomenon has been observed in aquaculture with a direct correlation observed between phage resistance and complete attenuation of *F. columnare* in a zebrafish system, such that one phage-sensitive phenotype resulted in 100% mortality compared to 0% in the phage-resistant phenotypes (Laanto et al., 2012).

In plant pathogens, there is also evidence to suggest that phage-mediated selection might alter the infectivity and/or virulence of bacterial pathogens. Phages infecting the bacterium, *Erwinia amylovora*, were found to be most efficient at infecting strains that produced either high or low levels of exopolysaccharides (depending on the phage family examined), suggesting strong and context-dependent selection on a trait that is also known to play a key role in virulence on the plant host (Roach et al., 2013). Furthermore, Hosseinidoust et al. (2013) found that in tissue culture, phage-resistant variants of *Pseudomonas aeruginosa* actually secrete higher levels of virulence factors and caused more damage to cultured mammalian cells. With no general pattern yet emerging, phage-mediated attenuation of virulence seems hard to predict and certainly not guaranteed. It is possible that a bacterial pathogen could evolve resistance to a phage therapy product and maintain or even attain high virulence levels. Furthermore, the role of compensatory mutations to phage resistance is unknown; in the case of antibiotics such mutations can rapidly ameliorate the costs paid for resistance (Levin et al., 2000; Brandis and Hughes, 2013) and the same may be true of resistance to phages. If this turns out to be the case, phage therapy treatments that rely on the loss of costly resistance (or interactions among costly mutations to multiple phages) will be at constant risk of bacterial escape.

## INCREASING HORIZONTAL GENE FLOW THROUGH PHAGE APPLICATION

In addition to the direct effects of phage application on the densities and relative frequencies of bacterial pathogens, we must also be aware of the potential dangers of phage-mediated horizontal gene transfer (HGT) among pathogenic and non-pathogenic bacterial species. This is particularly relevant as HGT is an important driver of antibiotic resistance evolution (Courvalin, 1994). Given that phages facilitate horizontal gene flow through the process of transduction and that beta-lactam resistance genes have been isolated from environmental phage genomes (Colomer-Lluch et al., 2011), there is clear need to be cautious in our application of phages in the environment. Moreover it has recently been shown that antibiotic treatment itself expands the number of genes that confer drug resistance in phage metagenomes (Modi et al., 2013). Environmental perturbation with antibiotics also expanded the ecological network of phages, suggesting that drug treatment selects for greater phage-mediated transfer of resistance genes (Modi et al., 2013). Finally, antibiotic treatment of swine increased the induction of prophage – another key mechanism of HGT (Allen et al., 2011).

Phage-mediated transfer of virulence factors is also a key concern, and has been well characterized in the *Vibrio cholerae* system whereby CTX Φ phage (among others) shapes the severity of cholera pandemics through the transfer of toxin producing virulence factors and environmental fitness benefits (Faruque and Mekalanos, 2012). Additionally, *V. cholerae* has been shown to become naturally competent on a chitin surface (similar to its environmental niche on crustacean exoskeletons), facilitating uptake of exogenous DNA from an array of sources (Meibom et al., 2005), which could be important if phages are lysing nearby pathogenic bacterial strains. Environmental perturbation through unnaturally high titers of phages could lead to high levels of transduction and horizontal gene flow with unintended outcomes, exacerbating antibiotic resistance and moving virulence factors and toxins among genomes. However, a better understanding of phage host range and host range expansion could help mitigate the spread of genes among bacterial hosts.

## IMPACT OF PHAGE APPLICATION ON NATURAL MICROBIAL COMMUNITIES

The importance of the microbial flora to the fitness of human hosts has become clear in recent years, with microbes playing a proposed role in obesity (Greenblum et al., 2012), oral health (Belda-Ferre et al., 2012), Crohn’s disease (Manichanh et al., 2006), AIDS (Saxena et al., 2012), and even mental health (Foster and McVey Neufeld, 2013). Although less well studied, a similar role of microbiota in shaping fitness is likely to be true for agricultural plants (Peiffer and Ley, 2013) and livestock (McFall-Ngai et al., 2013). Whilst antibiotics can disrupt a large proportion of the microbial community (Dethlefsen and Relman, 2011), phage therapy may facilitate the targeted elimination of a pathogenic strain without disruption of the normal microbiota of a patient (Carlton, 1999) or a crop plant. Indeed, this argument has been a focal one to support phage therapy over antibiotic use (Loc-Carrillo and Abedon, 2011). The term “dysbiosis” has been introduced to describe a microbial flora that has become “unhealthy,” typically in human disease (Tamboli, 2004). The same phenomenon is likely to hold true for natural microbial communities both within and outside of the host environments, and thus the addition of foreign phages in the form of a biopesticide could destabilize such communities, causing “dysbiosis” and potentially having subsequent effects on disease and nutrient cycling. For example, in soil where ratios of phages to bacteria are expected to be near 1:1 (Reyes et al., 2010) an influx of applied phages could well disrupt stable ecological communities important in nutrient cycling. Conversely, in the marine environment, where this ratio is closer to 1:100 (Reyes et al., 2010), an influx of applied phages is unlikely to adversely disrupt a normal microbial community. Unfortunately, by the same logic, aquaculture may suffer from lower chances of success from phage biocontrol, as the concentrations of phages needed to influence bacterial density is likely to be a limiting factor.

## UNPREDICTABILITY OF INFECTION KINETICS

Unlike antibiotic usage, where an effective dosage can be determined for different species and corresponding quantities used, the infection kinetics of phage therapy are less predictable (Payne and Jansen, 2003). This knowledge gap presents a challenge for effective use balanced with environmental safety, as an individual phage persists in the environment for relatively short timescales, but a lineage can reproduce indefinitely when hosts are plentiful. This continued replication makes calculations of dosage difficult. In antibiotic treatment the minimum inhibitory concentration (MIC) is crucial to informing proper antibiotic dosages; however, finding the right balance between a high enough titer of phages to be effective and not introducing excessive levels into the environment is difficult. Potentially the application of a single phage could lead to the continued replication of an infective phage, thus perpetuating not only treatment but also persistence in the environment. If there are unwanted effects of phage biopesticide there are no tools currently available to selectively remove such viruses from the environment. Also, the timing of treatment as a function of bacterial density can be crucial for a successful outcome (Payne and Jansen, 2003). Indeed, in a trial controlling *V. parahaemolyticus* infections in shrimp timing was crucial whereas changes in dosage had no effect. Reducing the multiplicity of infection made no difference whereas when treatment was delayed it was ineffective in controlling mortality (Martínez-Díaz and Hipólito-Morales, 2013), possibly as a result of non-linear infection kinetics. Such infection kinetics of phage therapy were tested *in vitro* with *Campylobacter jejuni*, a common poultry pathogen, and found to fit a non-linear model with a density-dependent proliferation threshold (Cairns et al., 2009). These studies highlight the nuances involved in effective phage therapy use and as such the use of phages cannot be analogous to that of antibiotics. Provided policy makers accept this and approach the field balancing the risks of widespread environmental use with the pressing need for a clinical alternative to antibiotics, phage biocontrol could be an integral tool in controlling bacterial diseases.

## FUTURE OUTLOOK

Our understanding of the biology, ecology, and evolution of microbial pathogens has improved immeasurably since the advent of widespread antibiotic use. If we can learn the lessons from our mistakes with antibiotic use, phage therapy and biocontrol could form an integral tool in the fight against bacterial infections that threaten human health and food production (Pirnay et al., 2012; Allen et al., 2013). For example, the falling costs of whole genome sequencing (Kisand and Lettieri, 2013) should make tracking the evolution and spread of resistance genes in a clinical setting easier and more accurate (Didelot et al., 2012). Furthermore, advances in metagenomics may make monitoring the effects of environmental perturbations on microbial communities feasible and allow researchers to track changes over long-time periods. An attractive avenue of research for pharmaceutical companies may be the use of phage lysins – enzymes that are capable of bursting bacterial cells open “from without.” Such an approach avoids the problems of infection kinetics mentioned previously and can have a broad-spectrum encompassing multiple strains of antibiotic resistant pathogens, such as MRSA and vancomycin intermediate *Staphylococcus aureus* (Gilmer et al., 2013). The downside is that lysins, unlike phages, lack the ability to counter evolve to pathogens.

Microbial biofilms present a continued risk to healthcare as they may harbor bacteria in a less metabolically active state that survive antibiotic treatment (Oliver, 2010). Phages targeting biofilms in synchrony with antibiotics may form a novel strategy, although the inherent risks of HGT still remain. It may also be possible to circumvent this cycle of treatment, selection for resistance and re-infection through the use of “social disruption” treatments that reduce bacterial virulence without selecting for resistance (Boyle et al., 2013). Phages could play a role in the reduction of biofilm and public good production – one example is an engineered phage that expresses a biofilm-degrading enzyme (Lu and Collins, 2007). Whilst this is likely to reduce the fitness of bacterial populations, selection should be weaker than that of an antibiotic. Given the difficulties faced by clinicians in treating antibiotic-resistant infections and the urgency of finding alternative therapies, the prudent use of phages should be a priority. Nevertheless we have the tools to track resistance and simple measures such as providing a diverse set of phages for treatment could help. A seasonal-vaccine-like scheme could create a treatment program that is responsive to the evolution of resistance. In short, a very different model to that of our use of antibiotics.

## Conflict of Interest Statement

The authors declare that the research was conducted in the absence of any commercial or financial relationships that could be construed as a potential conflict of interest.
